# The Biosynthesis of D-1,2,4-Butanetriol From d-Arabinose With an Engineered *Escherichia coli*

**DOI:** 10.3389/fbioe.2022.844517

**Published:** 2022-03-24

**Authors:** Jing Wang, Qiaoyu Chen, Xin Wang, Kequan Chen, Pingkai Ouyang

**Affiliations:** State Key Laboratory of Materials-Oriented Chemical Engineering, College of Biotechnology and Pharmaceutical Engineering, Nanjing Tech University, Nanjing, China

**Keywords:** D-1,2,4-butanetriol, D-arabinose, biosynthesis, metabolic engineering, bioengineering

## Abstract

D-1,2,4-Butanetriol (BT) has attracted much attention for its various applications in energetic materials and the pharmaceutical industry. Here, a synthetic pathway for the biosynthesis of BT from d-arabinose was constructed and optimized in *Escherichia coli*. First, *E. coli* Trans1-T1 was selected for the synthesis of BT. Considering the different performance of the enzymes from different organisms when expressed in *E. coli*, the synthetic pathway was optimized. After screening two d-arabinose dehydrogenases (ARAs), two d-arabinonate dehydratases (ADs), four 2-keto acid decarboxylases (ADXs), and three aldehyde reductases (ALRs), ADG from *Burkholderia sp*., AraD from *Sulfolobus solfataricus*, KivD from *Lactococcus lactis* IFPL730, and AdhP from *E. coli* were selected for the bio-production of BT. After 48 h of catalysis, 0.88 g/L BT was produced by the recombinant strain BT5. Once the enzymes were selected for the pathway, metabolic engineering strategy was conducted for further improvement. The final strain BT5Δ*yiaE*Δ*ycdW*Δ*yagE* produced 1.13 g/L BT after catalyzing for 48 h. Finally, the fermentation conditions and characteristics of BT5Δ*yiaE*Δ*ycdW*Δ*yagE* were also evaluated, and then 2.24 g/L BT was obtained after 48 h of catalysis under the optimized conditions. Our work was the first report on the biosynthesis of BT from d-arabinose which provided a potential for the large-scale production of d-glucose-based BT.

## Introduction

D-1,2,4-Butanetriol (BT) is a straight-chain non-natural four-carbon polyol with wide applications. In the military context, BT is the precursor of D-1,2,4-butanetriol trinitrate (BTTN) which can be used as a propellant and an energetic plasticizer (Niu et al., 2003). Compared to traditional nitroglycerine, BTTN is less hazardous, less shock-sensitive, less volatile, and more thermally stable (Niu et al., 2003). BT is also an important building block for the synthesis of several drugs with high value (Yamada-Onodera et al., 2007). It can be used as the precursor of a retardant that can control the release of a drug (Valdehuesa et al., 2014) and it can also be dehydrated to 3-hydroxytetrahydrofuran, a key component for the HIV drug *amprenavir* (Mandava et al., 2011). Currently, BT is mainly produced by the reduction of malic acid using NaBH_4_ as a reducing agent. This process will produce a large number of borate salts as by-products (Monteith et al., 1998). Besides this, the chemically synthesized butanetriol has two isomers that will limit its applications.

Due to the problems of the traditional chemical strategy, the microbial synthesis of BT was selected as an alternative route (Lu et al., 2016). In 2003, Niu et al. made the first report on the microbial synthesis of BT from d-xylose. By expressing d-xylose dehydrogenase, d-xylonate dehydratase, benzoylformate decarboxylase, and aldehyde reductase in *E. coli*, 1.6 g/L BT was obtained (Niu et al., 2003). After that, a series of strategies including screening enzymes with high activities; improving the activity of the rate-limiting enzyme; knocking out the branch pathway and so on, were applied to improve the conversion rate and concentration of BT. Jing et al. screened four decarboxylases from different organisms and the recombinant strain harboring the *kivD* gene produced 10.03 g/L BT (Jing et al., 2018); Sun et al. conducted systematic fine-tuning of the expression level of the enzymes and BT production was increased by 4.3-fold (1.58 g/L) from the prototype strain (Sun et al., 2016). Bamba et al. modified the metabolism of Fe^2+^ in *Saccharomyces cerevisiae* to improve the activity of d-xylonate dehydratase (XylD) which was considered as the rate-limiting enzyme, to enhance the synthesis of BT. Eventually, 1.7 g/L of 1,2,4-butanetriol was produced from 10 g/L xylose with a molar yield of 24.5% (Bamba et al., 2019). Metabolic engineering strategy was also conducted to disrupt the endogenous competitive pathways such as d-xylose isomerization pathway and 2-keto acid aldol pathway, for further improvement of the yield of BT from d-xylose in the past years (San et al., 2002; Abdel-Ghany et al., 2013; Zhang et al., 2016). The application of these strategies has made some progress in the biosynthesis of BT from d-xylose in these years. Besides the method mentioned above, BT can also be produced from d-glucose. In 2014, Li et al. reported a novel pathway for the biosynthesis of BT from d-glucose. d-glucose was first utilized by *E. coli* to produce malate which shares a similar structure with BT. Then, after six steps of catalysis, BT was successfully produced from malate. Finally, 120 ng/L BT was produced by *E. coli* using d-glucose as the sole carbon source (Li et al., 2014).

d-Glucose has been used for the industrial production of various bulk chemicals successfully (Yim et al., 2011; Zhang et al., 2011; Song et al., 2013). There is no doubt that achieving the production of BT from d-glucose is meaningful. However, the following obstacles make it hard to achieve the large-scale production of BT from d-glucose directly: First, the difficulty to achieve the perfect balance between cell growth, protein expression, and BT production; second, malyl CoA and 2,4-dihydeoxybutyryl CoA (two intermediates of the synthetic pathway from malate to BT) are not the natural substrates of succinate semialdehyde dehydrogenase (SucD) and Coenzyme A acylating aldehyde dehydrogenase (Ald). The metabolic flux is low; third, each molecule of BT needs to consume six molecules of cofactor (four NADH molecules and two ATP molecules) (Li et al., 2014). Thus, developing a more efficient method for the biosynthesis of d-glucose-derived BT is urgently needed. After screening various derivatives of d-glucose, d-arabinose attracted our attention as its structure is similar to d-xylose. d-Arabinose can be obtained by oxidizing d-gluconate with Fenton reagent (Maletzky and Bauer, 1998; Wang and Lemley, 2002), while d-gluconate has been produced in large quantities from d-glucose via a fermentation process (Znad et al., 2004). These reasons inspired us to develop a synthetic pathway to produce BT from d-arabinose, which can provide the production of d-glucose-derived BT with a biochemical method.

**FIGURE 1:**
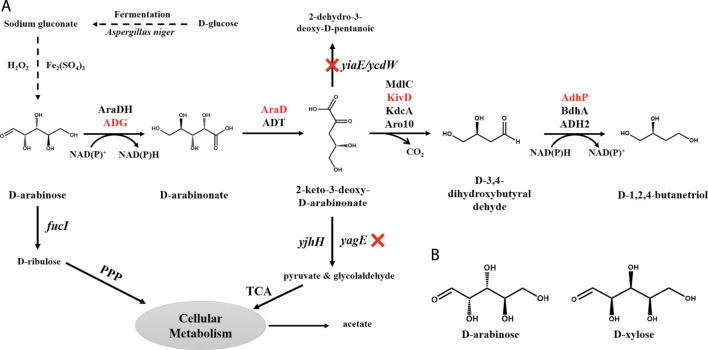
The BT biosynthetic pathway from d-arabinose in recombinant *E. coli*. **(A)** The dashed lines represent the synthetic method from d-glucose to d-arabinose. The synthetic pathway of BT and branch pathways are marked by solid lines. The red Xs indicate that the genes are knocked out and enzymes shown in red indicate that these enzymes have been chosen for the production of BT from d-arabinose after screening. Symbols: AraDH/ADG, d-arabinose dehydrogenase; AraD/ADT, d-arabinonate dehydratase; MdlC/KivD/KdcA/Aro10, 2-keto acid decarboxylase; AdhP/BdhA/ADH2, aldehyde reductase; *fucI* encoding l-fucose isomerase; *yiaE*/*ycdW* encoding glyoxylate reductase; *yagE*/*yjhH* encoding 2-keto-3-deoxy-d-arabinonate aldolase; PPP, pentose phosphate pathway; TCA, tricarboxylic acid cycle. **(B)** The structure of d-arabinose and d-xylose.

In this study, a synthetic pathway consisted of d-arabinose dehydrogenase (AraDH), d-arabinonate dehydratase (AraD), 2-keto acid decarboxylase (MdlC), and aldehyde reductase (AdhP) was designed and established in *E. coli* for the bio-production of BT from d-arabinose (Figure 1A). The host suitable for the pathway assembly with a higher activity of BT synthesis was first identified. Then, two ARAs, two ADs, four ADXs, and three ALRs from different organisms were evaluated to improve the production of BT. After that, the effect of by-product pathways on the biosynthesis of BT was also investigated. Under the optimized conditions, 2.24 g/L BT was produced after 48 h of bioconversion.

## Results and Discussion

### Designing a Novel Biosynthetic Pathway for BT Production in *E. coli*

At present, the *de novo* production of BT from d-glucose was achieved (Li et al., 2014). However, the imbalance between the cell growth, protein expression, and BT production, low enzyme activity, and huge demand for cofactors have resulted in the low BT production (120 ng/L) from d-glucose. d-Arabinose, the derivative of d-glucose, was used here to develop an alternative way to produce BT from d-glucose (Figure 1B). In previous studies (Liu et al., 2012; Valdehuesa et al., 2014; Lu et al., 2016; Sun et al., 2016; Bamba et al., 2019), BT was mainly obtained from d-xylose through a four-step catalytic reaction: dehydrogenation, dehydration, decarboxylation, and reduction (Wang et al., 2018; Bamba et al., 2019; Gao et al., 2019; Zhao et al., 2019; Yukawa et al., 2021). As the structure of d-arabinose is similar with d-xylose, a four-step synthetic pathway consisted of d-arabinose dehydrogenase, d-arabinonate dehydratase, 2-keto acid decarboxylase, and aldehyde reductase was accordingly conducted here to produce BT from d-arabinose (Figure 1A).

**FIGURE 2:**
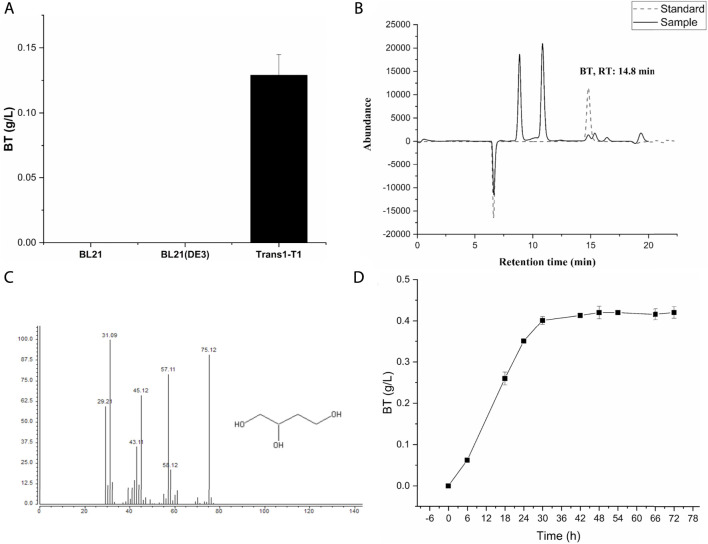
HPLC and GC-MS analysis for proof of the novel synthetic pathway for the production of BT from d-arabinose. **(A)** BT1 represents the strain *E. coli* Trans1-T1/pTrc99a-MdlC-AraDH, pCWJ-AraD-AdhP; BL21-1 represents the strain *E. coli* BL21/pTrc99a-MdlC-AraDH, pCWJ-AraD-AdhP; BL21 (DE3)-1 represents the strain *E. coli* BL21 (DE3)/pTrc99a-MdlC-AraDH, pCWJ-AraD-AdhP. The bioconversion process was carried out at 33°C on a rotatory shaker (200 rpm). **(B)** HPLC analysis for proof of the novel synthetic pathway to produce BT from d-arabinose. The solid line represents the abundance of standard BT and the dashed line represents the abundance of the sample. Retention time of BT was 14.08 min. **(C)** GC-MS analysis of the BT produced by strain BT1. **(D)** The time-course of the bio-conversion process catalyzed by the strain BT1. Bio-catalysis of d-arabinose to BT was conducted in a 100-ml Erlenmeyer flask which contains a 20-ml reaction mixture. OD_600nm_ of the reaction mixture was 60. The concentration of d-arabinose was 20 g/L. The titer of Mg^2+^ was 10 mM. The reaction mixture was incubated at 33°C on a rotatory shaker (200 rpm). Error bars represent SD (*n* = 3).

*E. coli*, the most widely used host for the production of various chemicals (Kaur et al., 2018), was used here for BT biosynthesis. AraDH (ARA from *S*. *solfataricus*) (Brouns et al., 2006), AraD (AD from *S*. *solfataricus*) (Brouns et al., 2006), MdlC (ADX from *P*. *putida*) (Tsou et al., 1990), and AdhP (ALR from *E. coli*) (Wang et al., 2018) were over-expressed in the *E. coli* strain BL21, BL21 (DE3), and Trans1-T1, respectively, to assemble the BT synthetic pathway from d-arabinose (Figure 1A). Then, these three strains were cultivated in LB medium and induced with 2 mM IPTG when OD_600nm_ of the culture reached 0.6. After incubating for 12 h, cells were harvested and used for the biosynthesis of BT from d-arabinose. After 24 h, 0.13 g/L BT was detected in the reaction mixture catalyzed by the whole-cells of BT1 (Figures 2A–C). Many factors affect the final yield of desired products in cells for the bioproduction process, especially for the unnatural product. There is no universal strategy to obtain a high yield directly. However, the screening of different hosts is a common, simple, and effective strategy for the successful production of unnatural product (Vuralhan et al., 2005; Falcioni et al., 2013; Wang et al., 2018). The reasons why the different host has a significant effect on the production of different product remains difficult to explain. The expression difference of heterologous proteins in different hosts might be one of potential factors. *E. coli* Trans1-T1 has been successfully used in the biosynthesis of various chemicals (Meng et al., 2015) and thus it was also used as a candidate here. As shown in Figure 2A, BT production was only successfully detected when *E. coli* Trans1-T1was used as the host. While none of BT was detected using the whole-cells of BL21-1 and BL21 (DE3)-1. SDS-PAGE analysis (Supplementary Figure S1) exhibited that insoluble expression of the d-arabinonate dehydratase (AraD) was found in *E coli* BL21-1 and BL21 (DE3)-1, which might be related with their failure on BT production. Hence, it was used for further research. Finally, a time-course of the bio-conversion was also conducted (Figure 2D), and the recombinant strain BT1 produced 0.42 g/L BT after 48 h of catalysis.

### Screening Enzymes for Improved BT Production

**FIGURE 3:**
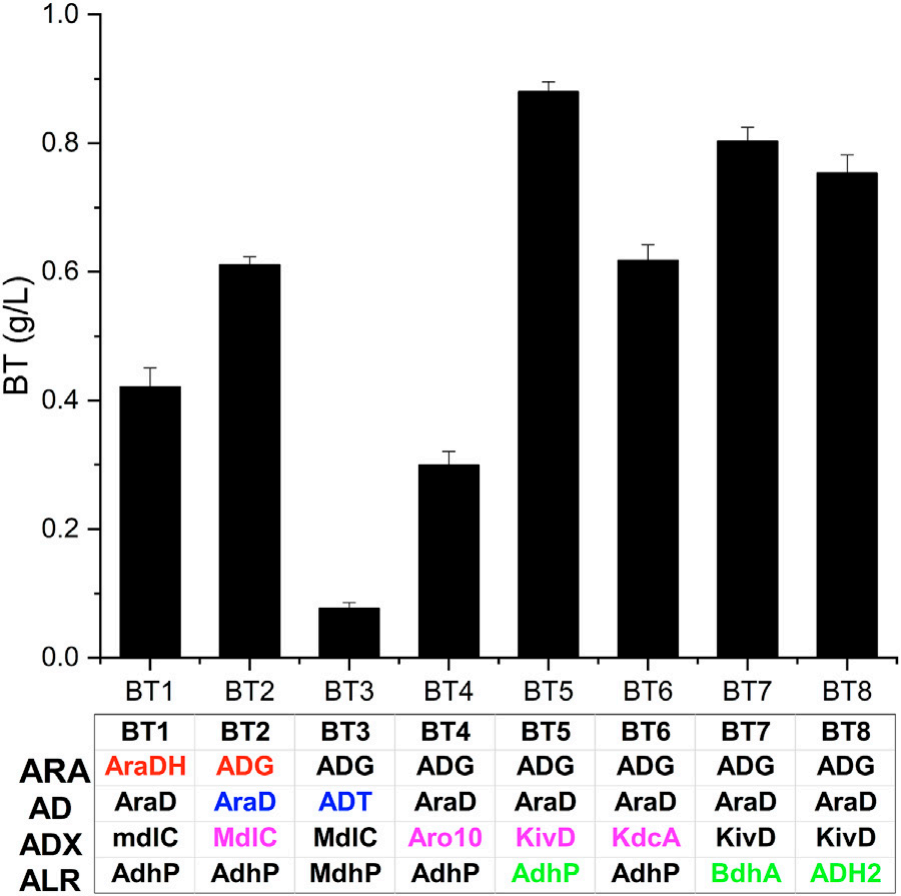
Screening enzymes for improved production of BT. BT1 and BT2 are used to evaluate the effect of ARA and enzyme names are shown in red; BT2 and BT3 are used to evaluate the effect of AD and enzyme names are shown in blue; BT2, BT4, BT5, and BT6 are used to evaluate the effect of ADX and enzyme names are shown in magenta; BT5, BT7, and BT8 are used to evaluate the effect of ALR and enzyme names are shown in green. Four enzymes expressed in each strain are listed in the table. Error bars represent SD (*n* = 3).

The BT biosynthetic pathway from d-arabinose consisted of four enzyme catalysis (dehydrogenation, dehydration, decarboxylation, and reduction). Considering that enzymes from various organisms may have different performances in BT production (Wang et al., 2018), we evaluated the effect of enzyme sources on the pathway efficiency. The pH value (pH 7.0) of the whole-cells of BT1 almost had no change during the production of BT. This may result from the low activity of the d-arabinose dehydrogenase encoded by *araDH*. Thus, ADG from *Burkholderia sp*. was over-expressed in BT2, compared with BT1 (0.42 g/L), the titer of BT catalyzed by whole-cells of BT2 reached 0.61 g/L improving 45%. In some reports focusing on the synthesis of BT from d-xylose, d-xylonate always accumulated and the dehydration reaction was considered as the rate-limiting step (Bamba et al., 2019; Bañares et al., 2019). Thus, we replaced AraD with ADT from *P*. *fluorescens* and constructed the recombinant strain BT3 for evaluating the effect of AD in the bio-production of BT. After 48 h, only 0.08 g/L BT was detected (Figure 3), which was only 13% of the activity of BT2. This result indicated that the activity of the d-arabinonate dehydratase was particularly important for the production of BT and AraD was more suitable than ADT in the synthesis of BT.

The third step of the synthetic pathway was catalyzed by 2-keto acid decarboxylase, a vital group of enzymes crucial to the production of keto acid derived alcohols (Atsumi et al., 2008). Thus, we applied another three ADXs: Aro10 from *S. cerevisiae*, KivD from *L*. *lactis* IFPL730, and KdcA from *L*. *lactis* B1157 to the biosynthesis of BT, respectively. As shown in Figure 3, recombinant strain BT5 expressing KivD produced 0.88 g/L BT which was 40% higher than that of BT2 expressing MdlC, proving that KivD was the most suitable ADX here. This result was consistent with the conclusion drawn by Jing et al. after screening four 2-keto acid decarboxylases in the production of BT from d-xylose (Jing et al., 2018). However, Wang et al. reported that KdcA from *L. lactis* B1157 performed best in the synthesis of BT (Wang et al., 2018). Different expression hosts and other enzymes used in the synthetic pathway may be responsible for this difference. The same situation happened in the evaluation of ALRs. In this research, BT5 expressing AdhP from *E. coli* performed better BT synthesis activity compared to BT7 expressing BdhA from *Bacillus subtilis* WB800N and BT8 expressing ADH2 from *S. cerevisiae* in the synthesis of BT (Figure 3). Although, Biswas et al. reported that overexpressing BdhA improved the production of 2,3-butanediol (Biswas et al., 2012), ADH2 was proved to perform well in the production of BT (Zhang et al., 2016). Wang et al. proved that AdhP was more suitable for the synthesis of BT after evaluating six ALRs (Wang et al., 2018). Here, the strain BT5 over-expressing ADG, AraD, KivD, and AdhP exhibited the best activity in the synthesis of BT from d-arabinose and the BT (0.86 g/L) production improved 105% compared to that of BT1 (0.42 g/L).

### The Effect of the By-Product Pathway on BT Synthesis

**FIGURE 4:**
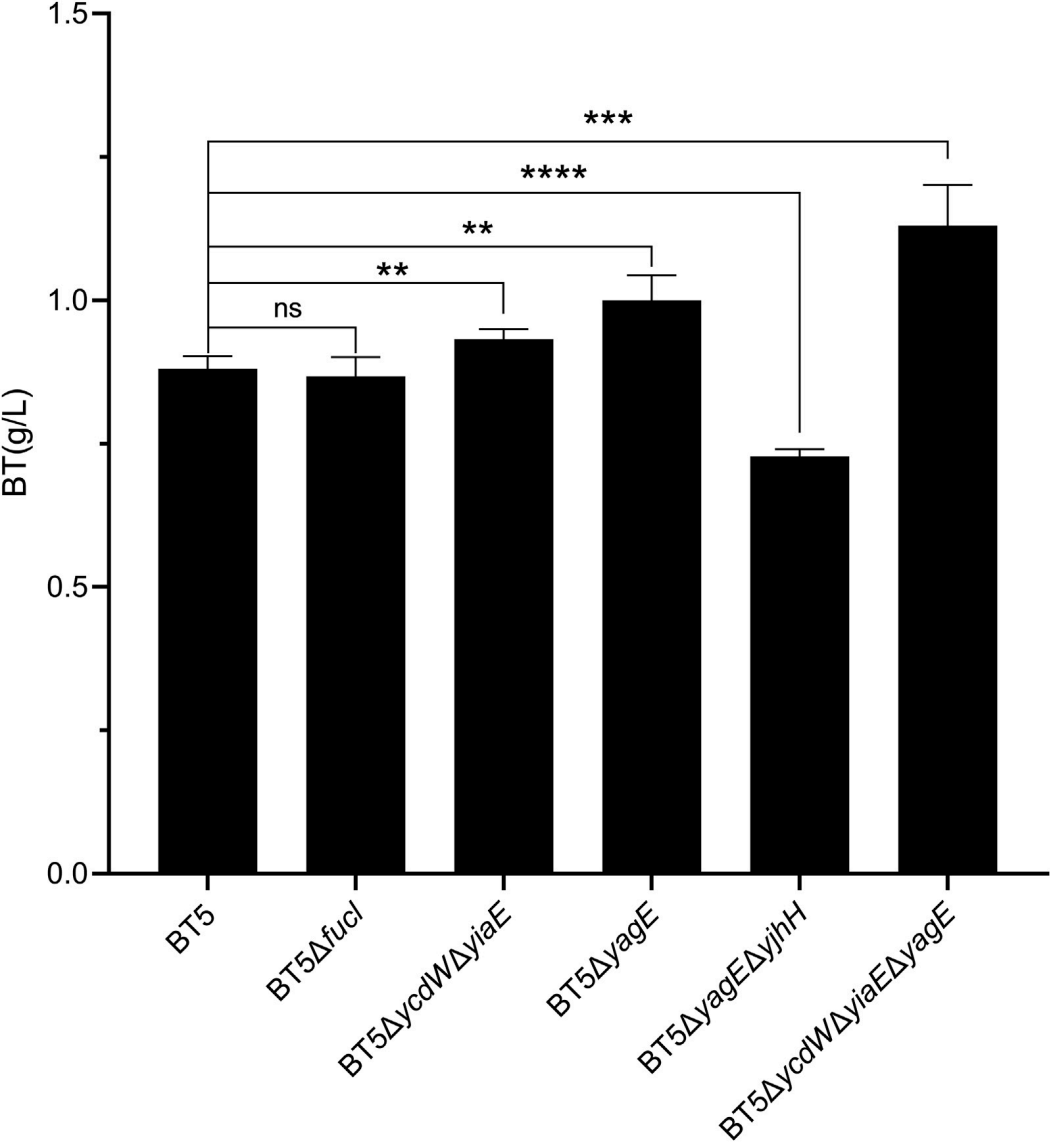
The BT produced by the metabolic engineered T1 series strains after catalyzing for 48 h. All of these six strains harbored the plasmid pCWJ-AraD-AdhP and pTrc99a-KivD-ADG. Statistical analysis was performed using Student’s t-test (two-tailed; *p < 0.05; **p < 0.01; ***p < 0.005; ****p < 0.001; ns, no significant difference). Error bars represent SD (*n* = 3).

In the pathway to produce D-1,2,4-butanetriol from d-xylose, the substrate and intermediates can be consumed by endogenous enzymes in *E. coli* (Sun et al., 2016; Jing et al., 2018; Bamba et al., 2019). Therefore, the engineering of byproduct pathway to improve the conversion yield of d-xylose to D-1,2,4-butanetriol has gained much attentions recently (Valdehuesa et al., 2014; Bamba et al., 2019; Bañares et al., 2019; Gao et al., 2019). The d-arabinose isomerase encoded by *fucI* of *E. coli* can catalyze the isomerization of d-arabinose to d-ribulose which may reduce the flux toward BT. Thus, the strain BT5Δ*fucI* was constructed. After 48 h catalyzed by the whole-cells of BT5Δ*fucI*, 0.87 g/L BT was produced which was almost the same as that of BT5 (Figure 4). This result was different from previous reports where disrupting the d-xylose isomerization pathway in *E. coli* improved the yield of BT (Valdehuesa et al., 2014; Jing et al., 2018). This difference might be associated with the fact that the *E. coli* host metabolizes d-xylose faster than d-arabinose (Supplementary Figure S2). Leblanc and Mortlock also reported that at least 5 days were needed before the growth of *E. coli* 1000 could be detected on d-arabinose (LeBlanc and Mortlock, 1971). The low consumption of d-arabinose by *E. coli* is undoubtedly beneficial for the synthesis of BT. After that, the gene *yiaE* and *ycdW* encoding the 2-keto acid reductase which was reported to catalyze the reduction of 2-keto acid (Jing et al., 2018) were knocked out, yielding the mutant strain BT5Δ*yiaE*Δ*ycdW*. As the results show in Figure 4, the strain BT5Δ*yiaE*Δ*ycdW* produced 0.93 g/L BT, which was 7% higher than that of BT5. In addition, 2-keto acid could also be converted to pyruvate and glycolaldehyde by native aldolase encoded by *yagE* and *yjhH* of *E. coli* (Valdehuesa et al., 2014). The strain BT5Δ*yagE* was constructed to evaluate its effect on the synthesis of BT from d-arabinose. After bioconversion of 48 h, 1.0 g/L BT was produced by the whole-cells of BT5Δ*yagE* with a 10% increase compared to that of BT5 (Figure 4). Afterward, we knocked out both *yagE* and *yjhH* genes to completely disrupt this branched pathway yielding the recombinant strain BT5Δ*yagE*Δ*yjhH*. Unfortunately, less BT (0.73 g/L) was produced by this strain. This was different from a previous report, simultaneously disrupting *yagE* and *yjhH* which encode the 2-keto acid aldolase promoted the synthesis of BT (Valdehuesa et al., 2014). As shown in Supplementary Figure S3, the cell density of the reaction conducted by the whole-cells of BT5Δ*yagE*Δ*yjhH* decreased faster than other groups within 24 h. This suggested that the reaction to generate pyruvate catalyzed by YagE and YjhH might be important to maintain the stability of the cells. Completely blocking the production of pyruvate was not conducive to the synthesis of BT. The final strain BT5Δ*yiaE*Δ*ycdW*Δ*yagE* produced 1.13 g/L BT after 48 h of catalysis.

### Optimizing Cultivation and Biotransformation Conditions for Improving BT Production

**FIGURE 5:**
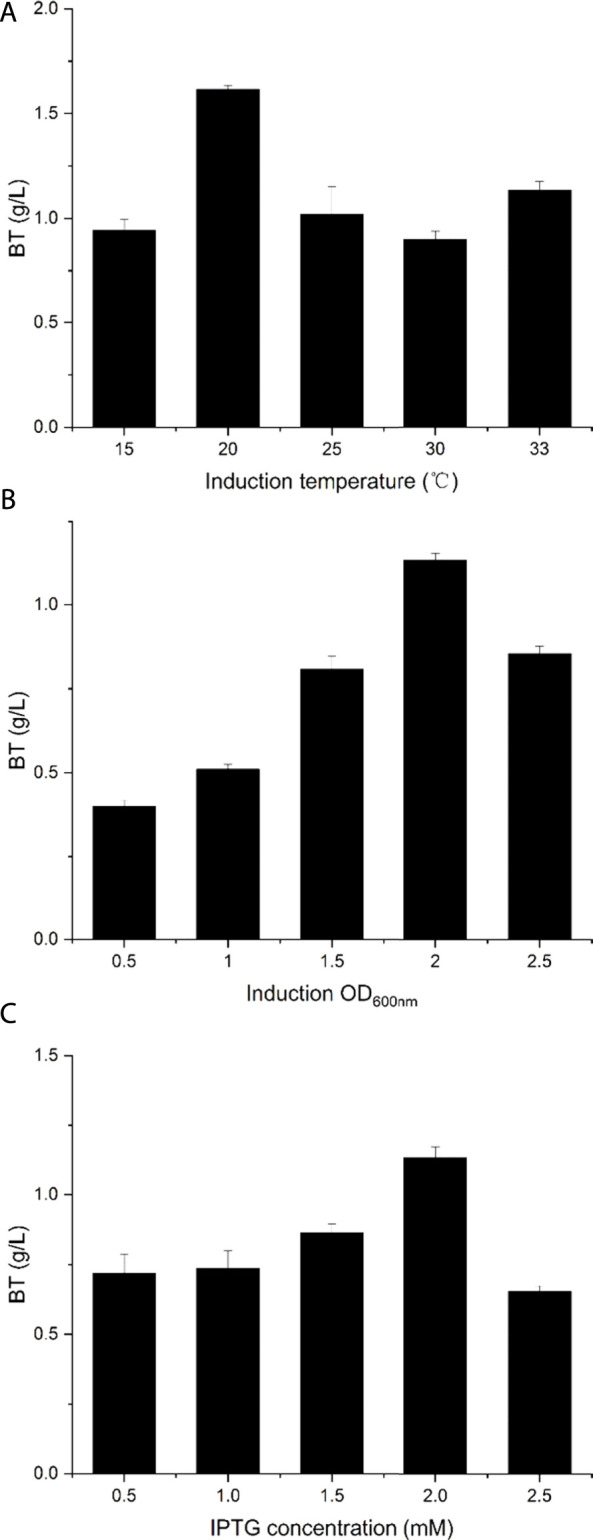
Optimizing the fermentation conditions to produce BT. **(A)** The optimum induction temperature. **(B)** The optimum induction OD_600nm_. **(C)** The optimum IPTG concentration. The original fermentation conditions: induction temperature was 33°C; induction OD_600nm_ was 2; IPTG concentration was 2 mM. Each experiment only changes a single variable. Error bars represent SD (*n* = 3).

It is well known that cultivation conditions contribute greatly to the recombinant pathway performance in *E. coli* and the production of d-xylose-derived BT had been increased after optimizing the fermentation conditions (Wang et al., 2018). Thus, the fermentation conditions including induction temperature, IPTG concentration, and induction OD_600nm_ were investigated to improve BT production. For the original fermentation condition, the IPTG concentration was 2 mM, the induction temperature was 33°C, and the induction OD_600nm_ was 2. As shown in Figure 5A, BT5Δ*yiaE*Δ*ycdW*Δ*yagE* was incubating at a temperature ranging from 15 to 33°C, and the maximum BT production was achieved when the strain was incubating at 20°C. After catalyzing for 48 h, 1.62 g/L BT was obtained, which improved 40% compared to that of the strain incubated at 33°C. The highest BT synthesis ability of BT5Δ*yiaE*Δ*ycdW*Δ*yagE* was gained when the induction OD_600nm_ was 2 (Figure 5B). Induction conducted at the middle phase of logarithmic growth reduced the damage caused by the over-expression of four enzymes (Supplementary Figure S4). Finally, the effect of the IPTG concentration was investigated by varying the titer from 0.5 to 2.5 mM. When adding 2 mM IPTG (final concentration), the strain exhibited the best catalytic activity and 1.13 g/L BT was achieved (Figure 5C). Compared to the strain induced with 0.5 mM IPTG, the activity increased by about 60%. Overall, under the optimal fermentation conditions (induction temperature was 20°C; induction OD_600nm_ was 2; IPTG concentration was 2 mM), the production of BT reached 1.62 g/L.

**FIGURE 6:**
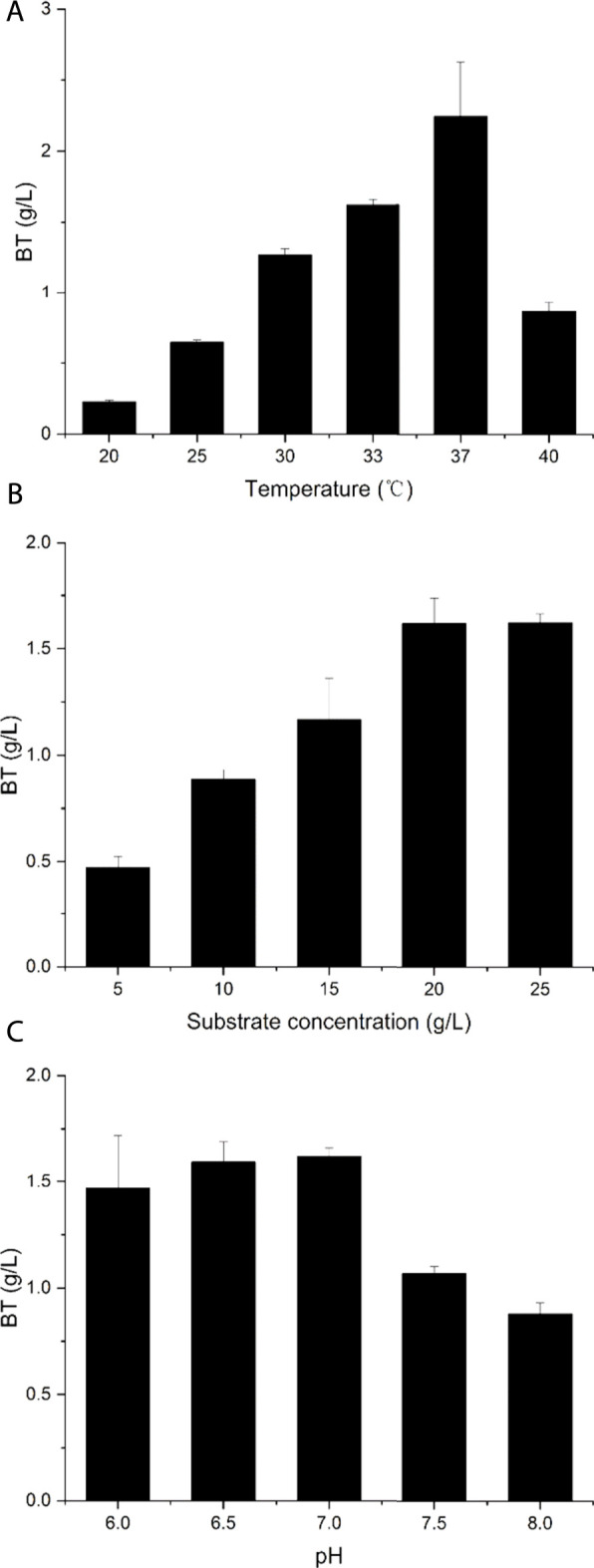
Characterization of the recombinant strain BT5Δ*yiaE*Δ*ycdW*Δ*yagE.* **(A)** The optimum catalytic temperature. **(B)** The optimum substrate concentration. **(C)** The optimum original reaction pH. The original catalytic conditions: reaction temperature was 33°C; substrate concentration was 20 g/L; reaction pH was 7.0. Each experiment only changes a single variable. Error bars represent SD (*n* = 3).

To further improve BT production, we also evaluated the bioconversion conditions by the recombinant strain BT5Δ*yiaE*Δ*ycdW*Δ*yagE*. Here, substrate concentration, catalytic temperature, and initial reaction pH were optimized for improved BT production. The initial reaction conditions were as follows: 20 g/L d-arabinose, pH 7.0, and 33°C. To determine the optimal reaction temperature, the reaction was carried out at 20°C, 25°C, 30°C, 33°C, 37°C, or 40°C, respectively (Figure 6A). From 20 to 37°C, the activity increased with the temperature rising and reached the maximum at 37°C, which was consistent with the result reported by Gao et al., in 2019 (Gao et al., 2019). As described in Figure 6B, the BT titer reached the highest level when the concentration of d-arabinose reached 20 g/L. The optimum initial reaction pH was 7.0 (Figure 6C), which was very close to the optimum pH for AraD (Brouns et al., 2006). Andberg et al. also reported this phenomenon (Andberg et al., 2016), and this result suggested that the dehydration reaction may be the vital point in the synthesis of BT. Under the optimal catalytic conditions, the production of BT reached 2.24 g/L. As mentioned earlier, during the production of BT from d-arabinose, the pH of the reaction mixture remained stable. This avoids the detrimental effect of a large pH drop when producing BT from d-xylose (Bañares et al., 2019). Compared with the recent reports on the biosynthesis of BT from d-xylose, the BT titer and yield produced from d-arabinose are not high enough (Jing et al., 2018; Yukawa et al., 2021). There are still many aspects that need to be improved to further improve the production of BT from d-arabinose: screening for more active dehydrogenases and dehydratases, fine-tuning the expression levels of each enzyme, and balancing the ratio of NAD(P)^+^/NAD(P)H.

The production of the by-products was also detected (Supplementary Figure S5). We found that the recombinant strain BT5Δ*yiaE*Δ*ycdW*Δ*yagE* produced 0.45 mM pyruvate, 27.8 mM acetate, and 7.9 mM ethylene glycol after bioconversion of 48 h. In the meantime, 50 mM d-arabinose remained in the reaction solution. This result indicated that acetate was the main by-product. In addition, the mass balance revealed that approximate 30 mM of d-arabinose was missed. This part of the substrate may exist as the intermediate product, or it may be consumed by the strain through an unreported metabolic pathway. Further research to evaluate the effect of blocking the acetate synthesis pathway on the production of BT or the metabolic process of d-arabinose in *E. coli* might be worthy to improve BT production.

## Conclusion

Here, the synthetic pathway for the biosynthesis of BT from d-arabinose was conducted in *E. coli* Trans1-T1. After screening two ARAs, two ADs, four ADXs, and three ALRs, ADG from *Burkholderia sp*., AraD from *S*. *solfataricus*, KivD from *L. lactis* IFPL730, and AdhP from *E. coli* were selected. After 48 h of catalysis, 0.88 g/L BT was produced by the strain BT5 expressing these four enzymes. Besides this, a metabolic engineering strategy was also employed in this work, the recombinant strain BT5Δ*yiaE*Δ*ycdW*Δ*yagE* produced 1.13 g/L BT after catalyzing for 48 h. Finally, fermentation conditions were optimized, and the recombinant strain BT5Δ*yiaE*Δ*ycdW*Δ*yagE* was also characterized. Under the optimized conditions, BT5Δ*yiaE*Δ*ycdW*Δ*yagE* produced 2.24 g/L BT after catalyzing for 48 h. Compared with d-xylose, *E. coli* consumes d-arabinose more slowly, which indicates that a higher conversion rate may be possible in the future. During the catalytic process, the catalytic rate of the substrate is slow, which may be caused by insufficient dehydrogenase activity. In the follow-up study, further screening of dehydrogenase is needed. Of course, in such a multi-enzyme catalyzed reaction process, the matching of the reaction rates of each step is also very important. A large and rapid synthesis of acid will cause a rapid drop in pH value, which is disadvantageous in the production of BT (Bañares et al., 2019). Acetate was found as the main by-product, and subsequent studies can evaluate the effect of the acetate synthesis pathway on the biosynthesis of BT. Overall, the work presented here offered an alternative biosynthesis pathway for the bio-production of BT. This paper was the first report on the biosynthesis of BT from d-arabinose and supplied a potential for the large-scale production of d-glucose-based BT.

## Methods

### Strains and Media

All the strains constructed in this study are listed in Table 1. The *E. coli* strain was cultured in Luria Bertani medium (tryptone 10 g/L, yeast extract 5 g/L, and NaCl 10 g/L) containing 50 mg/L Ampicillin and 50 mg/L Chloramphenicol. BT, β-d-1-thiogalactopyranoside (IPTG), MgSO_4_∙7H_2_O, Na_2_HPO_4_, KH_2_PO_4_, d-xylose, and d-arabinose were purchased from Aladdin Ind. Co., Ltd. (China).

**TABLE 1:** Strains used in this study.

Strains	Descriptions	References
Trans1-T1	F^−^φ80 (*lac*Z)ΔM15Δ*lac*X74 *hsd*R (r_k_^−^, m_k_^+^)Δ*rec*A1398*end*A1t*on*A	TransGen
BL21	*E. coli* B F^−^ *dcm omp*T *hsd*S (r_B_^-^m_B_^-^) *gal* [*mal*B^+^]_K-12_ (λ^S^)	TransGen
BL21 (DE3)	F^−^ *omp*T *hsd*S (r_B_^-^m_B_^-^) *gal dcm* (DE3)	TransGen
T1-1	Trans1-T1Δ*fucI*	This study
T1-2	Trans1-T1Δ*ycdW*Δ*yiaE*	This study
T1-3	Trans1-T1Δ*yagE*	This study
T1-4	Trans1-T1Δ*yagE*Δ*yjhH*	This study
T1-5	Trans1-T1Δ*ycdW*Δ*yiaE*Δ*yagE*	This study
BL21-1	BL21 harboring plasmid pCWJ-AraD-AdhP & pTrc99a-MdlC-AraDH	This study
BL21 (DE3)-1	BL21 (DE3) harboring plasmid pCWJ-AraD-AdhP & pTrc99a-MdlC-AraDH	This study
BT1	Trans1-T1 harboring plasmid pCWJ-AraD-AdhP & pTrc99a-MdlC-AraDH	This study
BT2	Trans1-T1 harboring plasmid pCWJ-AraD-AdhP & pTrc99a-MdlC-ADG	This study
BT3	Trans1-T1 harboring plasmid pCWJ-ADT-AdhP & pTrc99a-MdlC-ADG	This study
BT4	Trans1-T1 harboring plasmid pCWJ-AraD-AdhP & pTrc99a-Aro10-ADG	This study
BT5	Trans1-T1 harboring plasmid pCWJ-AraD-AdhP & pTrc99a-KivD-ADG	This study
BT6	Trans1-T1 harboring plasmid pCWJ-AraD-AdhP & pTrc99a-KdcA-ADG	This study
BT7	Trans1-T1 harboring plasmid pCWJ-AraD-BdhA & pTrc99a-KivD-ADG	This study
BT8	Trans1-T1 harboring plasmid pCWJ-AraD-ADH2 & pTrc99a-KivD-ADG	This study

### Construction of Plasmids

All the plasmids constructed in this study are listed in Table 2 and all primers used in this work are listed in Table 3. The genes: *araDH* and *araD* from *Sulfolobus solfataricus*, *aDG* from *Burkholderia sp*., *aDT* from *Pseudomonas fluorescens*, *bdhA* from *Bacillus subtilis* WB800N, and *aDH*2 from *Saccharomyces cerevisiae* were codon-optimized and synthesized by Sprin GenBioTech Co., Ltd. (Nanjing, China), respectively. The DNA fragment of *araDH* was inserted into *Nco*I/*Bam*HI sites of pTrc99a to yield the plasmid pTrc99a-AraDH. The DNA fragment of *araD*, *aDG*, and *aDT* was inserted into *Nco*I/*Bam*HI sites of pCWJ producing the plasmid pCWJ-AraD, pCWJ-ADG, and pCWJ-ADT, respectively. The fragment of *bdhA* and *aDH*2 was inserted between the *Nco*I and *Hind*III sites by in-fusion clone to generate the plasmid pCWJ-BdhA and pCWJ-ADH2. Then, the Trc-*araDH* fragment amplified from pTrc99a-AraDH with primer P1 and P2 was inserted into *Sac*I/*Bam*HI sites of pTrc99a-MdlC-XylB producing the plasmid pTrc99a-MdlC-AraDH. Primer P3 and P4 were used to amplify Trc-*araD* fragment and it was inserted into *Spe*I/*Kpn*I sites of pCWJ-YjhG-AdhP yielding the plasmid pCWJ-AraD-AdhP.

**TABLE 2:** Plasmids used in this study.

*E. coli* plasmids	Descriptions	References
pCWJ	Cm^r^, P_trc_, ori (RSF)	Lab stock
pTrc99a	Ap^r^, P_trc_, ori (pBR322)	This study
pCas	Kan^r^, P_araB_-*Red*, P_cas_-*Cas9*, repA101, ori (pSC101), P_lacIq_, *lacI*, P_trc_-sgRNA-pMB1	Lab stock
pTarget	Spe^r^, pJ23119, sgRNA, pMB1, *aadA*	Lab stock
pCWJ-YjhG-AdhP	Cm^r^, pCWJ harboring *yjhG* & *adhP*	Lab stock
pCWJ-AraD-AdhP	Cm^r^, pCWJ harboring *araD* & *adhP*	This study
pCWJ-ADT-AdhP	Cm^r^, pCWJ harboring *aDT* & *adhP*	This study
pCWJ-AraD-BdhA	Cm^r^, pCWJ harboring *araD* & *bdhA*	This study
pCWJ-AraD-ADH2	Cm^r^, pCWJ harboring *araD* & *aDH2*	This study
pTrc99a-MdlC-XylB	Ap^r^, pTrc99a harboring *mdlC* & *xylB*	Lab stock
pTrc99a-KivD-XylB	Ap^r^, pTrc99a harboring *kivD* & *xylB*	Lab stock
pTrc99a-KdcA-XylB	Ap^r^, pTrc99a harboring *kdcA* & *xylB*	Lab stock
pTrc99a-Aro10-XylB	Ap^r^, pTrc99a harboring *aro10* & *xylB*	Lab stock
pTrc99a-MdlC-AraDH	Ap^r^, pTrc99a harboring *mdlC* & *araDH*	This study
pTrc99a-MdlC-ADG	Ap^r^, pTrc99a harboring *mdlC* & *aDG*	This study
pTrc99a-Aro10-ADG	Ap^r^, pTrc99a harboring *aro10* & *aDG*	This study
pTrc99a-KivD-ADG	Ap^r^, pTrc99a harboring *kivD* & *aDG*	This study
pTrc99a-KdcA-ADG	Ap^r^, pTrc99a harboring *kdcA* & *aDG*	This study
pTarget-ΔfucI	Spe^r^, pJ23119, sgRNA-*fucI*, pMB1, *aadA*	This study
pTarget-ΔyiaE	Spe^r^, pJ23119, sgRNA-*yiaE*, pMB1, *aadA*	This study
pTarget-ΔycdW	Spe^r^, pJ23119, sgRNA-*ycdW*, pMB1, *aadA*	This study
pTarget-ΔyagE	Spe^r^, pJ23119, sgRNA-*yagE*, pMB1, *aadA*	This study
pTarget-ΔyjhH	Spe^r^, pJ23119, sgRNA-*yjhH*, pMB1, *aadA*	This study

sgRNA-*fucI*, sgRNA with an N20 sequence for targeting the *fucI* locus; sgRNA-*yiaE*, sgRNA with an N20 sequence for targeting the *yiaE* locus; sgRNA-*ycdW*, sgRNA with an N20 sequence for targeting the *ycdW* locus; sgRNA-*yagE*, sgRNA with an N20 sequence for targeting the *yagE* locus; sgRNA-*yjhH*, sgRNA with an N20 sequence for targeting the *yjhH* locus.

**TABLE 3:** Primers used in this study.

Name	Primers	Sequences (5’ - 3′)
P1	Trc-SacI-F	CGA​GCT​CTT​GAC​AAT​TAA​TCA​TCC​GGC​TCG
P2	AraDH-BamHI-R	CGG​GAT​CCT​TAC​GGG​GTG​ATA​A
P3	Trc-SpeI-F	CTA​GAC​TAG​TTT​GAC​AAT​TAA​TCA​TCC​GGC​TCG
P4	AraD-KpnI-R	GGG​GTA​CCT​TAA​GAT​TTG​CAT​TTG​TAT​TCT​TCG
P5	Trc-ADG-F	TTT​CTC​CGG​TTA​AAT​AAG​TCT​CCC​TTA​TGC​GAC​TCC​TGC​ATT​AGG
P6	Trc-ADG-R	GGT​CGA​CTC​TAG​AGG​ATC​GGA​TCC​TTA​ACG
P7	Trc-ADG-SacI-F	CGA​GCT​CTT​ATG​CGA​CTC​CTG​CAT​TAG​GAA​ATA​CT
P8	Trc-ADG-BamHI-R	CGG​GAT​CCT​TAA​CGA​CCG​AAA​GCG​TCA​GTA​CC
P9	Trc-ADT-F	TGC​ATT​AGG​AAA​TAC​TAG​ACT​CCT​GCA​TTA​GGA​AAT​ACT​AGT​TTG​ACA​AT
P10	Trc-ADT-R	GGA​TGA​TTA​ATT​GTC​AAG​TTA​GTG​AGA​GTG​ACG​CGG​AAC​TTC​AG
P11	Trc-bdhA-KpnI-F	GGG​GTA​CCT​TGA​CAA​TTA​ATC​ATC​CGG​CTC​G
P12	Trc-bdhA-SalI-R	ACG​CGT​CGA​CAT​TTG​TCC​TAC​TCA​GGA​GAG​C
P13	Trc-ADH2-KpnI-F	GGG​GTA​CCT​TGA​CAA​TTA​ATC​ATC​CGG​CTC​GTA
P14	Trc-ADH2-SalI-R	GCG​TCG​ACA​TTT​GTC​CTA​CTC​AGG​AGA​GCG​T
P15	Target-fucI-F	ATGTGCGTACCTACTGGTCAGTTTTAGAGCTAGAAATAGCAAGTT
P16	Target-fucI-R	TGACCAGTAGGTACGCACATACTAGTATTATACCTAGGACTGAGC
P17	Target-yiaE-F	TAC​CGC​TCG​TCG​GGT​TGT​GGGTT​TTA​GAG​CTA​GAA​ATA​GCA​AGT
P18	Target-yiaE-R	CCACAACCCGACGAGCGGTAACTAGTATTATACACTAGTATTATACCTAGGACTGAGC
P19	Target-ycdW-F	ACGCGTGGATGTTGCCAGAGGTTTTAGAGCTAGGTTTTAGAGCTAGAAATAGCAAGT
P20	Target-ycdW-R	CTCTGGCAACATCCACGCGTACTAGTATTATACCACTAGTATTATACCTAGGACTGAGC
P21	Target-yagE-F	CATGCTGCGCAGGTGGGCGAGTTTTAGAGCTAGTTTTAGAGCTAGAAATAGCAAGT
P22	Target-yagE-R	TCGCCCACCTGCGCAGCATGACTAGTATTATACACTAGTATTATACCTAGGACTGAGC
P23	Target-yjhH-F	CCGCAGAATACCGAAAACGAGTTTTAGAGCTAGTTTTAGAGCTAGAAATAGCAAGT
P24	Target-yjhH-R	TCGTTTTCGGTATTCTGCGGACTAGTATTATACCACTAGTATTATACCTAGGACTGAGC

The underlined part indicates the N20 sequence.

Plasmid pTrc99a-MdlC-AraDH was digested with *Bam*HI and *Sac*I and it was ligated with the fragment Trc-*aDG* which was amplified using the primer P5 and P6, by in-fusion clone, constructing the plasmid pTrc99a-MdlC-ADG. The fragment Trc-*araD* was removed from the plasmid pCWJ-AraD-AdhP after digestion with *Spe*I and *Kpn*I. After that, the vector part was ligated with Trc-*aDT* amplified with the primer P7 and P8 to generate the plasmid pCWJ-ADT-AdhP. The plasmid pTrc99a-Aro10-XylB, pTrc99a-kivD-xylB, and pTrc99a-KdcA-XylB was digested with *Sac*I and *Bam*HI, respectively, to remove the Trc-*xylB* sequence. Then, these linearized vector fragments were used to ligate with the Trc-*aDG* fragment amplified with the primer P9 and P10, respectively, to produce the plasmid pTrc99a-Aro10-ADG, pTrc99a-KivD-ADG, and pTrc99a-KdcA-ADG. Primer P11 and P12 were used to clone Trc-*bdhA* and this fragment was inserted into *Kpn*I/*Sal*I sites of pCWJ-AraD-AdhP yielding the plasmid pCWJ-AraD-BdhA. DNA fragment Trc-*aDH2* was amplified with primer P13 and P14, and the plasmid pCWJ-AraD-ADH2 was constructed in the same way. Primers P15/P16, P17/P18, P19/P20, P21/P22, and P23/P24 were used to amplify the pTarget series plasmid, respectively, yielding the plasmid: pTarget-ΔfucI, pTarget-ΔyiaE, pTarget-ΔycdW, pTarget-ΔyagE, and pTarget-ΔyjhH.

### Construction of the Mutant *E. coli* Strains

*E. coli*/Trans1-T1 (T1) competent cells harboring Cas9 were prepared as described (Pujol and Kado, 2000; Sharan et al., 2009). l-arabinose (10 mM final concentration) was added to induce the production of λ-Red and the electroporation process was conducted as described (Jiang et al., 2015). Primers used for the amplification of donor DNA were shown in Supplementary Table S1. Plasmid pTarget-Δ*fucI*, pTarget-Δ*yiaE*, and pTarget-Δ*yagE* were electroporated into T1 competent cells with corresponding donor DNA, respectively, yielding the mutant strain: T1Δ*fucI*, T1Δ*yiaE*, and T1Δ*yagE*. The elimination of pCas and pTarget series plasmids was conducted as described (Datsenko and Wanner, 2000). After curing pTarget and pCas series plasmids, mutant strain T1-1 and T1-3 were obtained. Then, the strain T1Δ*yiaE* was made into competent cells after the plasmid pTarget-Δ*yiaE* was cured. After that, the plasmid pTarget-Δ*ycdW* was co-transformed into the cells with donor DNA yielding the mutant strain T1Δ*yiaE*Δ*ycdW*. The mutant strain T1-2 was obtained after eliminating the plasmid pTarget-Δ*ycdW* and pCas. After curing the plasmid pTarget-Δ*yagE*, plasmid pTarget-Δ*yjhH* was co-transformed with donor DNA into strain T1Δ*yagE* harboring Cas9 and λ-Red yielding the mutant strain T1Δ*yagE*Δ*yjhH*. Mutant strain T1-4 was gained after curing pTarget and pCas series plasmids. Plasmid pTarget-Δ*yjhH* was also co-electroporated with donor DNA into T1Δ*yiaE*Δ*ycdW* competent cells containing Cas9 and λ-Red for the construction of strain T1Δ5 with gene *yiaE*, *ycdW,* and *yagE* disrupted.

### Culture Conditions

*E. coli*/Trans1-T1 series strains containing the plasmid of interest were cultured in 500 ml of LB medium added with 0.1 mM Ampicillin and 0.1 mM Chloramphenicol at 37°C on a rotatory shaker (200 rpm). When the optical density at 600 nm of the culture medium reached 0.6, IPTG (2 mM final concentration) and Mg^2+^ (10 mM final concentration) were added. After incubating at 33°C on a rotatory shaker (200 rpm) for 12 h, cells were harvested by centrifugation (6000 rpm for 15 min) and washed two times with deionized water.

### Biotransformation Conditions

Biocatalysis of d-arabinose to BT was conducted in a 20-ml phosphate buffer solution (pH 7.0, 12 g/L Na_2_HPO_4_, 3 g/L KH_2_PO_4_), which contained 20 g/L d-arabinose and recombinant *E. coli* cells (OD_600nm_: 60). The reaction mixture was incubated at 33°C on a rotatory shaker (200 rpm) for 48 h. After that, the reaction mixture was incubated at 33°C on a rotatory shaker (200 rpm) for 48 h. Samples were boiled for 5 min to stop the reaction and proteins were removed by centrifugation (12,000 rpm, 5 min). Finally, the supernatant was used for high-performance liquid chromatography analysis.

### Analytic Methods

The concentrations of d-arabinose and BT were analyzed as described (Jing et al., 2018). The detection of BT was conducted by gas chromatography-mass spectrometry (GC-MS, ISQ 7000, Thermo Fisher Scientific, Waltham, MA) equipped with a TG-5MS GC column (30 m × 0.25 mm × 0.25 μm), using the method described as follows. Five milliliters reaction mixture was centrifuged (12,000 rpm) for 5 min and the supernatant was pretreated at −80°C for 1 h. Then the sample was dried in a vacuum freeze dryer (0.12 mbar, −40°C) and washed by 1 ml methanol. After centrifuging (12,000 rpm) for 5 min, the supernatant was used for GC-MS analysis. A total of 0.2 μL sample was injected and the flow ratio was 100 using helium as the carrier gas. The inlet temperature, split flow, and purge flow was set at 300°C, 100 ml/min, and 5 ml/min, respectively. The oven temperature gradient program was set as follows: initially held at 50°C for 5 min, raised by 10°C/min to 200°C (held for 0 min), and finally increased to 300°C at 20°C/min (held for 5 min). The total run time was about 30 min. The MS conditions for identification of BT were as follows: full scan mode, 29–350 m/z mass-range. The ion source temperature was 280°C and EI was ionized at 70 eV.

## Data Availability

The original contributions presented in the study are included in the article/Supplementary Material, further inquiries can be directed to the corresponding author.

## Abbreviations

ARA, d-arabinose dehydrogenase; AD, d-arabinonate dehydratase; ADX, 2-keto acid decarboxylase; ALR, aldehyde reductase; BT, D-1,2,4-butanetriol.
