# Supplementary material for: The Biosynthesis of D-1,2,4-Butanetriol From d-Arabinose With an Engineered Escherichia coli
Source: Front Bioeng Biotechnol. 2022 Mar 24;10:844517. doi: 10.3389/fbioe.2022.844517 (PMC8989435; doi:10.3389/fbioe.2022.844517)
Supplement: Supplementary file 1 [file DataSheet1.docx]

**Supplementary material**

**The biosynthesis of D-1,2,4-butanetriol from D-arabinose in an engineered *Escherichia coli***

Jing Wang, Qiaoyu Chen, Xin Wang^*^, Kequan Chen, Pingkai Ouyang

State Key Laboratory of Materials-Oriented Chemical Engineering, College of Biotechnology and Pharmaceutical Engineering, Nanjing Tech University, Nanjing 211816 Jiangsu, China

*Corresponding authors: Xin Wang

Email: [xinwang1988@njtech.edu.cn](mailto:xinwang1988@njtech.edu.cn)


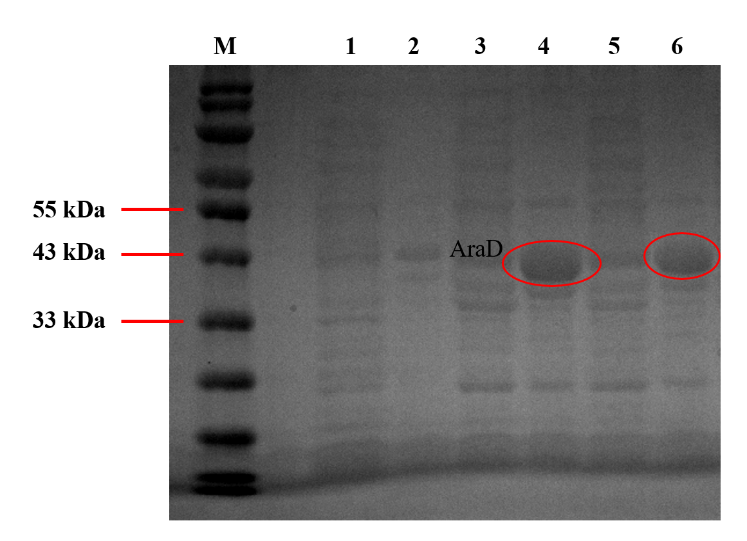


Fig. S1. SDS-PAGE analysis of fractions of the strain BT1, BL21-1, and BL21(DE3)-1. Intracellular fraction was prepared on ice by ultrasonication: 20 min pulsing (0.3 ms, 0.2 ms pause) at 40% input power and insoluble fraction of the lysate was removed by centrifugation (12000 rpm, 10 min). In soluble fraction was washed twice by deionized water before analysis. Samples (25 μg) were analyzed by electrophoresis on a 12% polyacrylamide gel after incubating at 100 °C for 10 min. M, protein marker (*Blue Plus*^®^ V) purchased from TransGen Biotech Co., Ltd. (China). Line 1, soluble protein from T1-1; Line 2, insoluble protein from T1-1; Line 3, soluble protein from BL21-1; Line 4, insoluble protein from BL21-1; Line 5, soluble protein from BL21(DE3)-1; Line 6, insoluble protein from BL21(DE3)-1.





Fig. S2. The amount of D-arabinose and D-xylose used by *E. coli*/Trasn1-T1 in 48 h. A 20 mL reaction mixture containing 20 g/L D-arabinose or D-xylose was incubated at 37 °C on a rotatory shaker (200 rpm). The cell density of the reaction mixture was 60 (OD_600nm_). Error bars represent SD (n=3).





Fig. S3. The change of the cell density during the bio-conversion process. The bio-conversion conditions were as follows: original OD_600nm_ was 60, D-arabinose concentration was 20 g/L, original pH was 7.0, reaction temperature was 37 °C. Error bars represent SD (n=3).





Fig. S4. The final cell density after 12 h of incubation. The strain BT5Δ5 over-expressing *ADG*, *AraD*, *kivD*, and *adhP* was induced with 2 mM IPTG at 33 °C on a rotatory shaker when OD_600nm_ reached 0.6 and 2 respectively. Blank presents the cell density of the strain BT5Δ5 without induction after 12 h. Error bars represent SD (n=3).





Fig. S5. By-products produced by recombinant strain BT5Δ5 after 48 h of catalysis. Error bars represent SD (n=3).

**Table S1. Primers used for the preparation of donor DNAs**

| **Primers** | **Sequence (5’- 3’)** |
| --- | --- |
| fucI-donor-frag1-F | atcccagcattctccatttacgg |
| fucI-donor-frag1-R | gtaaggaagcggcgttgtagccaagtgattcttcc |
| fucI-donor-frag2-F | gctacaacgccgcttccttaccgaaggc |
| fucI-donor-frag2-R | ttaacgcttgtacaacggaccgtag |
| yiaE-donor-frag1-F | acgtgattggttataccgacagc |
| yiaE-donor-frag1-R | cgggcgttgaagcaaaaattgctgcattttgttcgac |
| yiaE-donor-frag2-F | gcaatttttgcttcaacgcccgctactg |
| yiaE-donor-frag2-R | ttagtccgcgacgtgcgg |
| ycdW-donor-frag1-F | tggtggattgaggcactgcg |
| ycdW-donor-frag1-R | cctgatgctttaccggcaaagctttgcac |
| ycdW-donor-frag2-F | agctttgccggtaaagcatcaggattcctgctatccttggc |
| ycdW-donor-frag2-R | gcggctgcttcagcaacg |
| yagE-donor-frag1-F | GACTGCACCGAGCCATCTT |
| yagE-donor-frag1-R | ATCCAGCTGAGCGACGATCGACATGATCGATAG |
| yagE-donor-frag2-F | CGATCGTCGCTCAGCTGGATACGCCGTTTGTGAAC |
| yagE-donor-frag2-R | CCCAATGCCCGGTGTGGTA |
| yjhH-donor-frag1-F | CCTTCGGGGATGGGCCATA |
| yjhH-donor-frag1-R | AGCACGTGGACTTAAAACAGCCCGTCGACCCC |
| yjhH-donor-frag2-F | GGGCTGTTTTAAGTCCACGTGCTGCTTACC |
| yjhH-donor-frag2-R | TTCGTAATGCCCCTGGTGCA |
